# P-511. Preliminary results of clinical signs and brain imaging findings among neonates with Congenital Cytomegalovirus identified through the U.S. Surveillance for Emerging Threats to Mothers and Babies Network (SET-NET), 2014-2023

**DOI:** 10.1093/ofid/ofaf695.726

**Published:** 2026-01-11

**Authors:** Sarah B Mulkey, Nicol Awadalla Bacon, Kyle Spagnolo, Samantha Distler, Kate Russell Woodworth, Christina Sancken, Kelley Raines, Tatiana Lanzieri, Lexie Barber, Jacinda Merrill, Kathryn Aveni, Nicole D Longcore, Jane Fornoff, Kimberly Noble Piper, Van Tong

**Affiliations:** Children's National Hospital/ George Washington University School of Medicine and Health Sciences, Washington, DC; Children's National Medical Center, Washington, District of Columbia; Children's National Hospital, Washington, District of Columbia; CDC, A, Georgia; CDC, A, Georgia; CDC, A, Georgia; Centers For Disease Control And Prevention, Atlanta, Georgia; Centers for Disease Control and Prevention, Atlanta, Georgia; Minnesota Department of Health, St. Paul, Minnesota; Utah Department of Health and Human Services, Salt Lake City, Utah; New Jersey Department of Health, Trenton, New Jersey; New York State Department of Health, Albany, New York; Illinois Department of Public Health, Springfield, Illinois; Iowa Dept. Health and Human Services, Des Moines, Iowa; U.S. CDC, Chamblee, Georgia

## Abstract

**Background:**

Congenital cytomegalovirus (cCMV) is the most common infectious cause of birth defects and non-genetic hearing loss in U.S. children. Variations in cCMV screening practices and the absence of national cCMV surveillance in the U.S. hinders assessment of disparities in disease burden, clinical care, and intervention. We describe clinical characteristics of neonates with cCMV identified through a novel surveillance program.

**Table 1.**
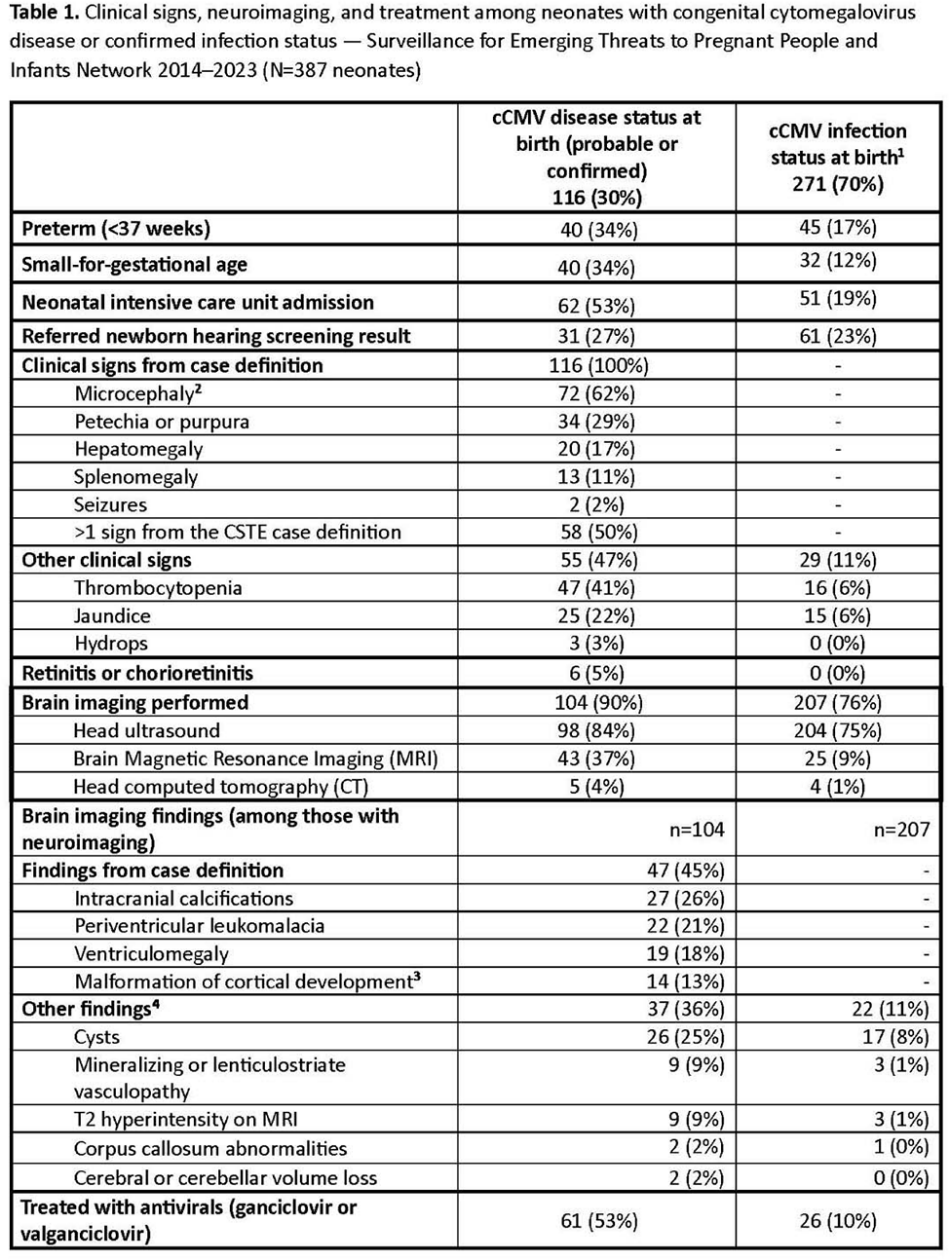
Clinical signs, neuroimaging, and treatment among neonates with congenital cytomegalovirus disease or confirmed infection status — Surveillance for Emerging Threats to Pregnant People and Infants Network 2014–2023 (N=387 neonates) 1 Some neonates classified as having cCMV infection at birth may go on to develop signs and symptoms later (e.g., sensorineural hearing loss, cerebral palsy) which would move them to a cCMV disease classification. Follow up is ongoing, and these results are preliminary. Additionally, 60% of infection cases came from Minnesota where a universal screening program is in place. 2Reported in the medical record or a reported head circumference <3 standard deviations below the mean for age and sex using INTEGROWTH-21st standards, ICD-10-CM code of Q02 or mention of microcephaly in the medical record. 3Malformation of cortical development includes cortical dysplasia, lissencephaly, pachygyria, polymicrogyria, or schizencephaly. 4Intraventricular hemorrhage and findings reported as directly related to intraventricular hemorrhage were not included.

**Figure 1.**
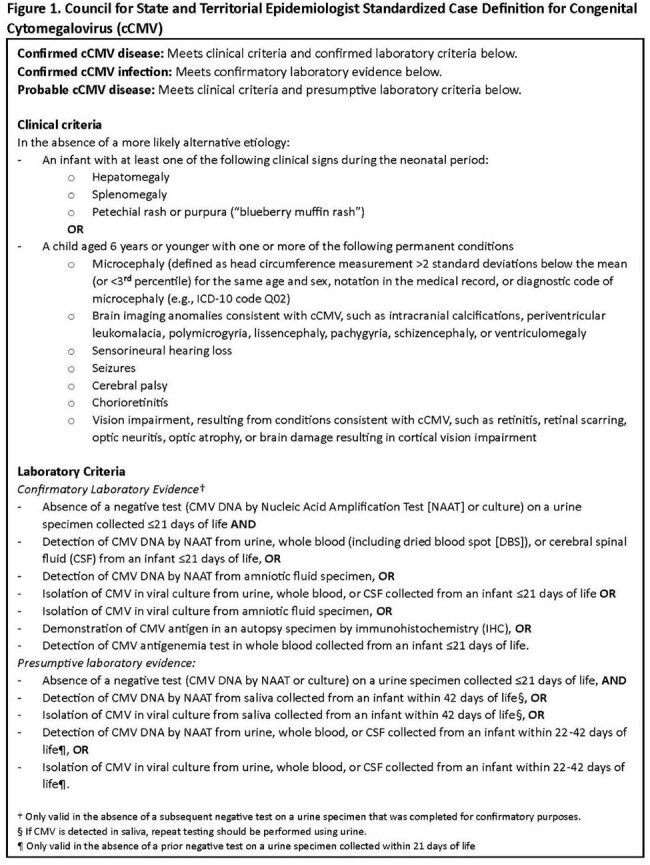
Council for State and Territorial Epidemiologist Standardized Case Definition for Congenital Cytomegalovirus (cCMV)

**Methods:**

Six states (Illinois, Iowa, Minnesota, New Jersey, New York state [excluding New York City], and Utah) ascertained neonates with cCMV through jurisdiction-specific screening practices (universal [n=1], hearing-targeted [n=3], high-risk [n=1], diagnostic codes [n=1], and clinical report [n=2]), including births from 2023 (5 states) or 2014 to 2023 (1 state). Case classification followed the 2023 Council of State and Territorial Epidemiologists surveillance case definition (Figure 1). Data were collected through vital statistics, laboratory reports, and birth hospitalization medical records.

**Results:**

Among 387 neonates with completed medical record abstraction, 73% were classified as confirmed cCMV infection, 28% confirmed cCMV disease, and 2% probable cCMV disease (Table 1). The most commonly reported signs among neonates with cCMV disease were microcephaly (62%) and petechia/purpura (29%), and the most common brain abnormalities among those with imaging (n=104) were intracranial calcifications (26%) and leukomalacia (21%). Of infants with cCMV infection, 25% had other signs reported not within the disease case criteria (e.g., small-for-gestational-age, brain cysts). Overall, 22% (87/387) were reported to have started treatment with antivirals within the first 14 days of life, including 26 with cCMV infection.

**Conclusion:**

These preliminary surveillance data demonstrate the feasibility of conducting cCMV surveillance. These findings should be interpreted with caution given the likelihood of under-ascertaining those with infection or with cCMV-associated signs not in the screening criteria. Preliminary findings are informing ongoing state efforts to improve surveillance and inform outreach to providers and caregivers. Continued surveillance and longitudinal data collection will provide more information on disease burden.

**Disclosures:**

Sarah B. Mulkey, MD, PhD, Pfizer: Advisor/Consultant

